# A Longitudinal Study of Health Improvement in the Atlanta CHDWB Wellness Cohort

**DOI:** 10.3390/jpm4040489

**Published:** 2014-12-22

**Authors:** Rubina Tabassum, Lynn Cunningham, Emily Hope Stephens, Katelyn Sturdivant, Gregory S. Martin, Kenneth L. Brigham, Greg Gibson

**Affiliations:** 1 School of Biology, Georgia Institute of Technology, Atlanta, GA 30332, USA; E-Mails: rubina.tabassum@helsinki.fi (R.T.); emilystephens30@gmail.com (E.H.S.); ksa.sturdivant@gmail.com (K.S.); 2 Institute for Molecular Medicine Finland, University of Helsinki, Helsinki FI-00014, Finland; 3 Emory University School of Medicine, Atlanta, GA 30308, USA; E-Mails: LCUNNIN@emory.edu (L.C.); greg.martin@emory.edu (G.S.M.); KBRIGHA@emory.edu (K.L.B.)

**Keywords:** personalized medicine, health partner, chronic disease risk, lifestyle intervention

## Abstract

The Center for Health Discovery and Wellbeing (CHDWB) is an academic program designed to evaluate the efficacy of clinical self-knowledge and health partner counseling for development and maintenance of healthy behaviors. This paper reports on the change in health profiles for over 90 traits, measured in 382 participants over three visits in the 12 months following enrolment. Significant changes in the desired direction of improved health are observed for many traits related to cardiovascular health, including BMI, blood pressure, cholesterol, and arterial stiffness, as well as for summary measures of physical and mental health. The changes are most notable for individuals in the upper quartile of baseline risk, many of whom showed a positive correlated response across clinical categories. By contrast, individuals who start with more healthy profiles do not generally show significant improvements and only a modest impact of targeting specific health attributes was observed. Overall, the CHDWB model shows promise as an effective intervention particularly for individuals at high risk for cardiovascular disease.

## 1. Introduction

Chronic non-communicable disorders (NCDs) are the leading cause of mortality worldwide. The 2010 report of the Global Burden of Diseases study (GBD2010) showed that in the United States, cardiovascular disease (CVD), cancer, musculoskeletal disorders, depressive disorders and diabetes are all among the top 10 sources of the burden of disease [1,2]. Globally, CVD is the leading cause of death and ischemic heart disease is the leading cause of disability-adjusted life years (DALYs) [1]. Since NCDs affect many aspects of life, including mortality, longevity, sense of wellbeing, work efficiency, and absenteeism, efforts should be made to help high-risk individuals shift their clinical profiles to more optimum health levels.

A large percentage of CVDs and NCDs could be prevented by reduction in behavioral risk factors such as unhealthy diets, physical inactivity, and tobacco use [3]. These unhealthy behaviors cause metabolic and physiological changes which lead to increase in metabolic risk factors such as high blood pressure, high blood glucose and high cholesterol. High blood pressure is the major risk factor for DALYs and leading cause of mortality associated with CVD (13% of global health). The behavioral risk factors which contribute significantly to the total mortality or DALYs due to CVDs are largely preventable and thus promotion of healthy lifestyles, which in effect may reduce metabolic risks, should be an important component in efforts to improve health and well-being.

The Center for Health Discovery and Well Being (CHDWB) at Emory University in Atlanta, Georgia USA, is one initiative aiming towards the prevention of the chronic diseases through promotion of healthy lifestyles. The CHDWB was established in 2008 with the intent to create a health-focused program that provides preventive care for healthy individuals through personalized interventions based on state-of-the-art self-knowledge. The center generates physical, physiological, biochemical, psychological and lifestyle profiles of all participants, who are generally healthy employees drawn at random from all sectors of Emory University. Based on the profiles at the time of recruitment, health partners of the Center provide personalized interventions, namely counselling regarding promotion of a healthier lifestyle. These may include changes in diet or exercise regimen, suggestions for stress reduction, or more specific interventions, but there is no attempt to prescribe a common diet plan, to specify a uniformly optimal fitness target, or to mandate changes: all health behavior changes (if any) are entirely at the discretion of the individual. Participants were examined at baseline, approximately six and 12 months later, and annually thereafter. An initial report on a small subset of this cohort (51 participants) already demonstrated the promises of the CHDWB program [4]. This initial analysis showed statistically significant reductions in BMI, percentage of body fat and systolic blood pressure, as well as increased fitness and serum high density lipoprotein-cholesterol (HDL-C) concentration in just six months. Improvement in cardiovascular and mental health was also observed. In the succeeding five years, the Center has enrolled a total of 668 participants, of whom 502 participants completed their six-month evaluation, 382 completed the one-year evaluation, and 83 have completed a total of five visits.

With this longitudinal clinical profile data, we set out to establish whether the CHDWB health promotion model achieved the objective of reducing the risk factors for NCDs and CVDs. We asked: (1) whether risk factors for NCDs decreased over the first three visits; (2) whether the sign and magnitude of change in profile was a function of baseline level; (3) whether the co-variance of risk factors over time identifies distinct sub-sets of individuals who show coordinated health improvements; and (4) to what extent priorities identified by each participant match observed shifts in their personally relevant clinical measures. The study reports consistently significant overall improvement in the physical, cardiovascular and mental health of the participants, as well as reduction in risk factors for CVD and other NCDs, that are stably maintained over three years. Participants towards the high end of the spectrum of risk for CVD at baseline showed the greatest improvements and tendency to reduce risk factors, and these individuals accounted for most of the overall change in health profiles. Although the mechanism remains unclear and we do not find concrete evidence that health partner relationships are responsible for the benefits, our data provides evidence that initiatives such as the CHDWB can promote preventive care and reduce the risk factors in an “essentially healthy” population.

## 2. Results and Discussion

### 2.1. Results

**Figure 1:**
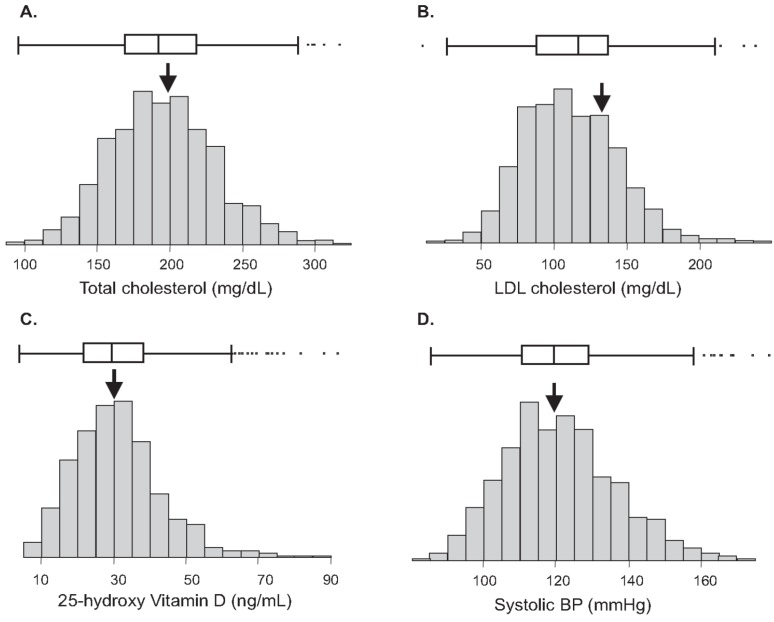
Distribution of cardiovascular risk factors in The Center for Health Discovery and Wellbeing (CHDWB) cohort at baseline. Arrows indicate reference mean for US population at large. Box and whisker show 1 and 2 standard deviations from the mean. Frequency distribution histograms are for (**A**) Total cholesterol, (**B**) LDL cholesterol, (**C**) Vitamin D, and (**D**) systolic blood pressure.

Since recruitment targeted “essentially healthy” participants, the CHDWB cohort represents a healthy population relative to the characteristics of the general US population. Individuals were excluded if they were functionally impaired by poorly controlled chronic disease or acute illness, but included if taking medications for common ailments. Demographic and clinical characteristics of CHDWB participants at baseline are provided in Appendix Table A1. Prevalence of obesity, hypertension and diabetes in US were reported to be 35.7%, 28.6% and 8.2% in 2010 [5]. The CHDWB cohort had lower prevalence of these chronic NCDs compared to the general US population. Of the total 668 participants in the CHDWB cohort, which includes 217 men and 451 women with a mean age of 48 years, 28.4% were obese subjects, 22.1% had hypertension and 3.6% had diabetes at baseline. Though the prevalence of clinical disease is relatively low, a significant proportion of individuals were nevertheless at pre-clinical risk of NCDs, particularly CVD. Almost half of the participants had abnormal systolic blood pressure (>120 mm/Hg) and total cholesterol levels >200 mg/dL (Figure 1), both of which are central risk factors for CVD [6]. Vitamin D level, which has already been shown to be associated with risk of CVD in the CHDWB cohort [4], was also lower than normal (<30 ng/mL) in more than 50% of the participants. Likewise, other risk factors for NCDs including BMI, percent body fat, LDL-C, and depression were at elevated levels in the cohort.

**Figure 2:**
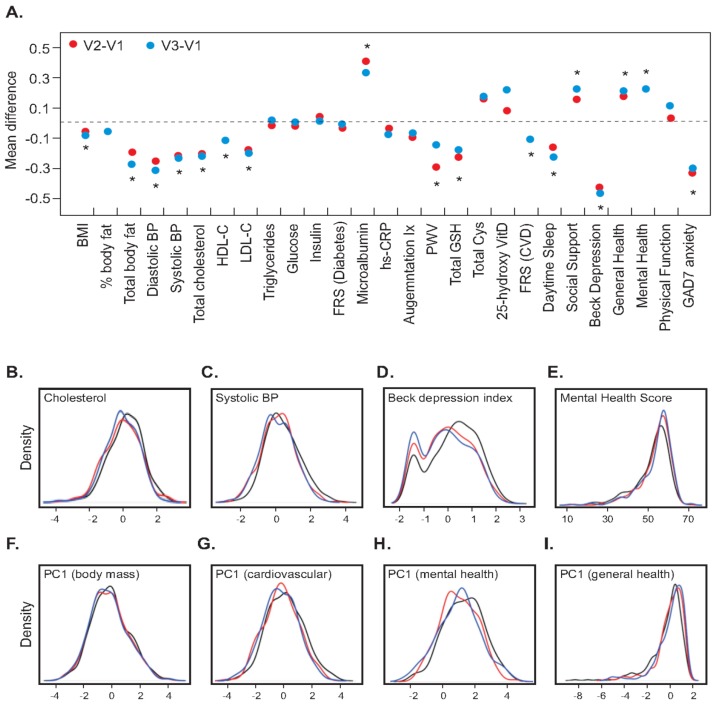
Changes in health measures in first three visits. (**A**) Plot of mean differences between Visit 2 (red) or Visit 3 (blue) and Baseline (Visit 1) for the indicated standardized trait measures. Asterisks indicate that the Visit 2 difference was significant at *p* < 0.0005 by Wilcoxon paired sample rank sign test; (**B**–**E**) Kernel density plots of the frequency of individuals with the indicated values for four clinical measures at Baseline (black), Visit 2 (red) and Visit 3 (blue), in each case showing left-ward shift of the curve (rightward for the Mental Health score) with fewer individuals with unhealthy measures; (**F**–**I**) Kernel density plots for Principal Component 1 summary measures for multiple contributing traits in each of the four domains, as described in the methods, also implying a general shift toward improved health in the cohort as a whole.

The distribution of clinical traits and biochemical profiles in the cohort suggests that a large proportion of apparently healthy individuals are at high risk of developing CVDs and could be high priority targets for prevention and management of chronic disorders. With this goal, each participant discussed individualized health-promotion behaviors with their health partners, often targeting diet and physical activity regimes based on their clinical profile. If, for example, someone wanted a health plan that included double the normal fat intake, the health partner would have discouraged this, but if the participant really wanted that as their health plan, then that is what it was. Importantly, there were no prescribed targets commonly applied to the whole cohort. Occasionally participants were referred to an appropriate clinic if required. Participants were examined again after six months, and approximately at one year intervals. Table 1 summarizes characteristics of 382 participants, all of whom had completed three visits. The characteristics of the subjects who completed three visits were similar to those who did not attend the follow-up examinations. Significant improvement in the health of participants, particularly, cardiovascular and mental health, was observed during the first six months and this was continued or maintained for at least one year. Very similar results were observed for the full set of 502 participants for the contrast of Visit 1 and Visit 2 (not shown). As assessed by paired Wilcoxon signed rank tests, there were significant shifts in median levels of blood pressure, total cholesterol, LDL-C, 25-hydroxyvitamin D, mental health and depression, in all cases towards the optimal range from baseline to second visit and again to third visit (*p* < 0.05 for all comparisons). Figure 2B–E shows kernel density plots contrasting baseline (black), 6-month (red) and 12-month (blue) scores for four representative traits which showed significant shifts towards more optimal values over the first three visits. Overall, the cohort has shown marked improvement in the health and all shifts (with the exception of HDL-Cholesterol) were in the desirable direction.

**Table 1:** Characteristics at baseline and follow-up examinations, with comparison across the visits.

Trait	Mean ± S.D.			*p* Value		
	Visit 1	Visit 2	Visit 3	V1/V2	V1/V3	V2/V3
BMI	27.37 ± 5.89	27.11 ± 5.71	26.95 ± 5.75	3.1 × 10^−6^	2.5 × 10^−8^	0.12
Fat %	32.33 ± 9.5	31.97 ± 9.78	31.95 ± 9.79	0.02	0.04	0.57
Total body fat	27.21 ± 9.14	25.79 ± 8.63	25.42 ± 8.42	7.5 × 10^−11^	2.6 × 10^−13^ *	0.004
Diastolic BP (mmHg)	76.26 ± 10.46	73.90 ± 9.66	73.85 ± 10.5	4.7 × 10^−8^ *	1.5 × 10^−9^ *	0.81
Systolic BP (mmHg)	120.58 ± 15.74	116.72 ± 14.61	115.78 ± 14.06	3.0 × 10^−9^ *	1.5 × 10^−14^ *	0.22
Total cholesterol (mg/dL)	196.65 ± 35.91	189.7 ± 36.7	188.44 ± 32.95	6.3 × 10^−6^ *	2.1 × 10^−9^ *	0.72
HDL-C (mg/dL)	64.16 ± 17.98	62.00 ± 17.76	62.29 ± 17.86	1.2 × 10^−4^	4.3 × 10^−5^	0.85
LDL-C (mg/dL)	111.93 ± 31.77	107.00 ± 31.44	105.42 ± 29.58	2.3 × 10^−4^ *	1.9 × 10^−7^ *	0.36
Triglycerides (mg/dL)	103.94 ± 64.88	104.42 ± 58.81	104.03 ± 59.63	0.85	0.82	0.17
Glucose (mg/dL)	89.70 ± 18.29	90.64 ± 16.96	90.75 ± 31.97	0.97	0.69	0.09
Insulin	8.03 ± 8.35	7.90 ± 6.15	9.03 ± 17.17	0.64	0.88	0.82
FRS (Diabetes)	1.85 ± 3.73	2.39 ± 5.44	1.89 ± 3.94	0.56	0.66	0.49
Microalbumin	0.59 ± 1.4	0.84 ± 2.18	0.75 ± 1.25	2.0 × 10^−10^ *	7.6 × 10^−10^ *	0.71
hs-CRP (mg/dL)	0.37 ± 0.39	0.39 ± 0.41	0.39 ± 0.43	0.34	0.16	0.67
AIX	21.46 ± 9.79	21.30 ± 9.74	20.55 ± 10.02	0.04	0.08	0.54
PWV (m/s)	7.29 ± 1.43	7.01 ± 1.34	7.08 ± 1.19	5.0 × 10^−6^ *	3.7 × 10^−4^ *	0.17
Total GSH (uM)	4.55 ± 1.60	4.22 ± 1.45	4.17 ± 1.17	1.2 × 10^−4^ *	0.012 *	0.43
Total Cys (uM)	182.68 ± 37.97	189.03 ± 39.42	190.32 ± 37.40	0.0015 *	0.002 *	0.038
25-hydroxyvitamin D (ng/mL)	31.62 ± 12.81	31.73 ± 11.69	33.84 ± 11.02	0.59	1.7 × 10^−4^ *	0.16 *
FRS (CVD)	6.07 ± 6.38	5.23 ± 4.65	5.21 ± 5.06	8.0 × 10^−6^	1.0 × 10^−7^	0.99
Day-time Sleep Score	6.77 ± 4.04	6.06 ± 3.8	5.84 ± 3.84	4.6 × 10^−5^ *	1.4 × 10^−8^ *	0.21
Social Support Score	27.91 ± 5.04	28.8 ± 4.73	29.17 ± 4.61	3.2 × 10^−7^ *	7.7 × 10^−11^ *	0.78
Depression Score	5.3 ± 5.45	3.51 ± 4.54	3.25 ± 4.3	3.4 × 10^−11^ *	8.5 × 10^−11^ *	0.05
General Health Score	53.04 ± 7.85	54.53 ± 7.34	54.65 ± 7.47	3.7 × 10^−7^ *	3.6 × 10^−8^ *	0.29
Mental Health Score	52.23 ± 7.64	53.97 ± 6.79	54.41 ± 7.73	7.2 × 10^−7^ *	5.0 × 10^−7^ *	0.23
Physical Function Score	53.15 ± 5.49	53.41 ± 5.6	53.97 ± 5.46	0.02	5.0 × 10^−8^ *	8.0 × 10^−4^ *
Anxiety Score	3.38 ± 0.50	2.35 ± 2.75	2.41 ± 3.21	3.1 × 10^−7^ *	1.0 × 10^−4^	0.44

Data for 382 participants who completed at least three visits are presented. *p* Values obtained from paired Wilcoxon test performed on Z-scores of log transformed values. * *p* Value <0.05 for unpaired Wilcoxon test.

Next, we calculated the average change in standardized z-scores for each trait for each individual from baseline to six months and one year. Representative traits, most of which showed significant changes, are plotted in Figure 2A. The mean Framingham Cardiovascular Risk Score (FRS-CVD, a commonly used predictor of risk of heart attack calculated from an individual’s age, gender, total and HDL cholesterol, blood pressure and smoking status: http://cvdrisk.nhlbi.nih.gov) shifted from 6.1% to 5.2% in one year, with a significant change of −0.11 z-score units (*p* = 1.0 × 10^−7^) (Figure 2A). Pulse Wave Velocity (PWV), another measure of cardiovascular health [7], reduced significantly at six months (change = −0.3 z-score units, *p* = 5.0 × 10^−6^) but then showed a non-significant increase. Reductions in CVD risk factors including BMI, lipids and blood pressure were also observed. However, traits related to glucose metabolism including plasma glucose and insulin levels, and consequently FRS-Diabetes (similar to FRS-CVD but also incorporating BMI, fasting glucose and triglyceride levels as well as family history of diabetes) remained unchanged (Table 1 and Figure 2). Significant changes in emotional (social support) and psychological factors (depression and anxiety) were also seen. The first principal component analysis of factors related to cardiovascular disease, obesity, depression, physical and mental health, also changed in the desired direction, as shown in the kernel density plots in Figure 2F–I. Each indicates a reduction in the proportion of individuals with highly positive obesity, CVD, and depression scores, or negative mental health scores, reiterating our finding that there is significant improvement in physical, cardiovascular and mental health of the participants after 6 months, that either continued to improve or was at least maintained for one year.

**Figure 3:**
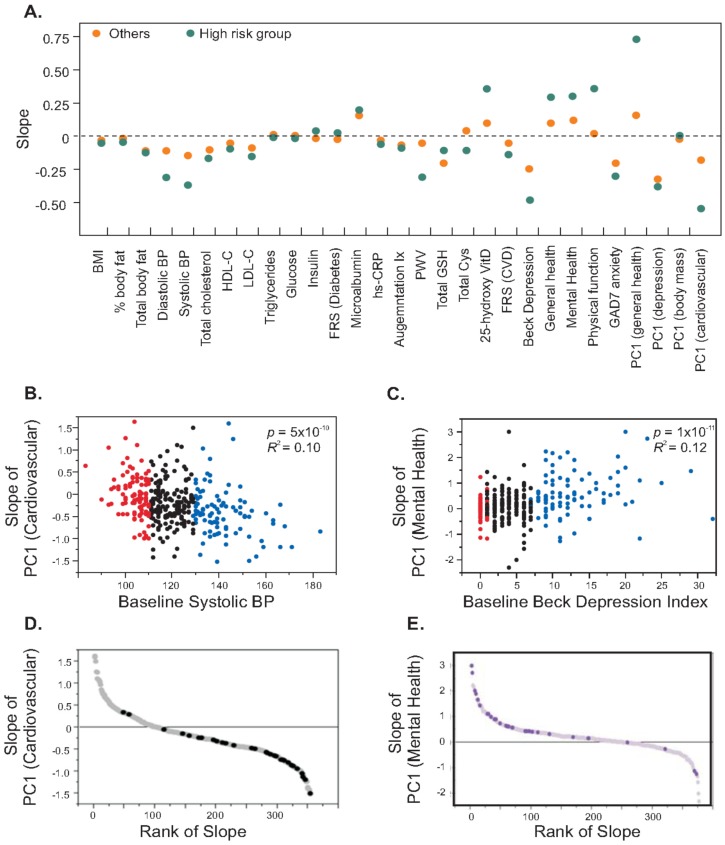
Health improvement in individuals with high CVD risk and low mental and physical health. (**A**) The plot contrasts the slope for the indicated traits for individuals in the high risk quartile of the trait (green), and the remaining three quarters of the cohort (orange). For 14 of the traits there is a significant difference with the change in the desired direction for all but total glutathione (Total GSH); (**B**,**C**) x-y plots of the slope of PC1 for cardiovascular risk factors or mental health respectively, against baseline systolic blood pressure or Beck depression index. Red and blue colors mark individuals in the bottom and top quartiles. A negative slope for PC1 (cardiovascular) implies improving health, and is greater for individuals with high blood pressure at baseline. A positive slope for PC1 (Mental Health) implies improved health, and is greatest for individuals who were more depressed at baseline; (**D**,**E**) Plots of the respective slopes by rank, with individuals at combined highest risk of physical and mental disease (in both less healthy quartiles) highlighted, showing enrichment for strong health responses in the desired direction.

A second striking observation is that the change in risk factors was highly correlated with their baseline levels, where the participants who had higher risk factors tended to show more improvement compared to participants who were relatively healthier at the initiation visit. We illustrate the relationship between baseline levels and slope generated from three visits for each trait in Figure 3A. These observations hold both for individual traits, and for individual traits relative to principal component (PC1) scores that summarize correlated traits. Principal components are descriptive statistics that capture the covariance of multiple traits and are often used to discover common tendencies, but here we use PC1 as a quantitative measure of traits known a priori to represent domains of health such as cardiovascular, metabolic, or mental health. For example, the left panel of Figure 3B shows a strong negative correlation between baseline systolic blood pressure and slope of principal component for each of the FRS-CVD risk factors. Categorization of subjects based on high systolic blood pressure (≥75th percentile) and low systolic blood pressure (≤25th percentile) generated a highly significant difference in slope of the principal component for CVD risk factors (*p* = 6.0 × 10^−7^), since individuals having high systolic blood pressure tended to have negative slopes (average = −0.37) while the group with low initial blood pressure did not show any change in slope (average = 0.01). Similarly, individuals with higher baseline depression score showed increases in physical and mental health while those with lower depression score remained unchanged (Figure 3C). We identified a high risk group from the cohort (N = 31) as those individuals having high CVD risk (≥75th percentile) and low mental and physical health (≤25th percentile) and Figure 3D and Figure 3E show that 95% of subjects in the high risk group showed a reduction in the risk of CVD and 87% of subjects showed improvement in mental and physical health. These analyses suggested that subjects having more risk factors tend to show more improvement than relatively healthier subjects.

We also investigated the relationships between health assessment scores as obtained by surveys using standard questionnaires and biochemical/clinical measures of disease risk, particularly CVD, to see how well they reflect the health status of the individual. General Health Score was correlated with HDL-C, glucose, triglycerides, FRS (Diabetes), 25-hydroxyvitamin D and cholesterol/HDL-C ratio (*p* < 1.0 × 10^−4^) (Table 2). Physical Function score also correlated with measures of cardiovascular diseases including augmentation index, sub-endocardial vascular risk ratio (SEVR) and systolic blood pressure (*p* < 1.0 × 10^−4^). Physical Function score correlated with levels of 25-hydroxyvitamin D and triglycerides which are known to be associated with CVD (Table 2). Thus, there is a good degree of concordance in health assessment scores and biochemical/clinical measures of cardiovascular health and risks. These scores comprise components which are easily modifiable and preventable, and thus could be used as non-invasive markers to assess health risks.

**Figure 4:**
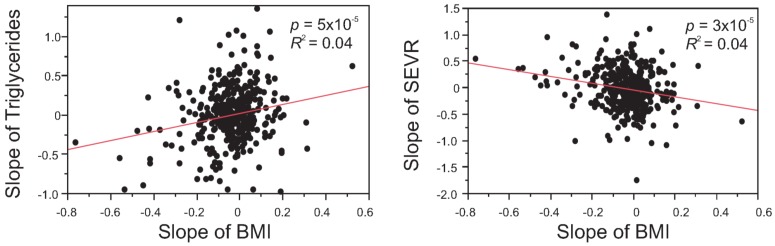
Correlation between the slopes of risk factors. (**A**) Significant positive correlation between change in BMI and change in Triglycerides, largely driven by individuals whose BMI drops (negative slope) also showing reduced triglycerides; (**B**) Significant negative correlation between slope of sub-endocardial vascular risk ratio (SEVR) and slope of BMI, again indicating improved cardiovascular health in individuals whose BMI decreases overall. *p*-Values for Pearson correlations are indicated on the figure, corresponding Spearman rank correlations are (**A**) 0.15 (*p* = 3.6 × 10^−3^) and (**B**) −0.19 (*p* = 2.8 × 10^−4^).

To investigate whether these scores also indicate improvement in cardiovascular function, we tested the relationship between slopes of traits to identify the factors which vary together (Appendix Table A2). We did not find any significant correlation between the health assessment scores and biochemical/clinical measures or risk factors for CVD. However, the slope of BMI showed the expected negative correlation with the slope of the general health score. Furthermore, the slope of BMI was also correlated with the slopes of risk factors for CVD and diabetes (Figure 4), suggesting that decrease in BMI is associated with general improvement in health. It is apparent, though, that these correlations are strongly dependent on the most extreme individuals, and that for the majority of participants there is not a significant correlated response in health measures.

**Table 2:** Relationship between health assessment scores and biochemical measures in the entire CHDWB cohort (N = 668) at baseline.

Health Assessment Score	Biochemical Trait	Pearson Correlation Coefficient	Spearman Correlation Coefficient	Pearson *p*-Value
General Health Score	HDL-C	0.20	0.23	3.1 × 10^−7^
	25-hydroxyvitamin D	0.15	0.19	7.8 × 10^−5^
	FRS (Diabetes)	−0.25	−0.27	2.1 × 10^−10^
	Triglycerides	−0.20	−0.22	1.6 × 10^−7^
	Cholesterol/HDL-C	−0.20	−0.23	1.7 × 10^−7^
	Glucose	−0.16	−0.13	5.4 × 10^−5^
Physical Function Score	SEVR	0.24	0.27	8.9 × 10^−10^
	25-hydroxyvitamin D	0.19	0.16	5.2 × 10^−7^
	FRS (Diabetes)	−0.26	−0.27	2.0 × 10^−11^
	Cysteine	−0.26	−0.27	1.5 × 10^−11^
	Glucose	−0.21	−0.16	5.9 × 10^−8^
	Systolic B.P.	−0.18	−0.19	3.9 × 10^−6^
	Triglycerides	−0.17	−0.14	8.0 × 10^−6^
	FRS (CVD)	−0.16	−0.16	3.0 × 10^−5^
	Microalbumin	−0.16	−0.12	5.4 × 10^−5^
	AIX	−0.16	−0.11	7.3 × 10^−5^

Abbreviations: HDL-C: high density lipoprotein-cholesterol; FRS: Framingham risk score; AIX: augmentation index; CVD: cardiovascular disease; SEVR: sub-endothelial vascular risk ratio.

In order to assess whether individuals are more likely to improve in health parameters that they self-identify as of high priority, we examined data for a subset of 162 participants for whom notes on discussions with their health-partner (with identities blinded to the research team) were available along with relevant measures, for at least four visits. Table 3 lists the most common priorities, the number of individuals for whom they were a concern, as well proportion of those individuals for whom the slope of change was in the desired direction, the opposite direction, or unchanged (adopting an arbitrary one quarter of a standard deviation unit in either direction as a threshold for non-neutral slopes). The last two columns compare the percentage of these participants in the self-prioritizing category and the remainder of the subjects in the sample. With the exception of BMI, the trends are all in the direction of a greater response when individuals self-prioritize the trait, but either the difference in proportions or the total sample size is too small to achieve statistical significance in each case. Most notably, body weight (*p* = 0.01, Fisher’s exact test, not corrected for multiple comparisons), stress and sleep quality may all improve when targeted by the participant.

**Table 3:** Targeted Health Responses in 162 Participants.

Prioritized Trait	Number	Threshold	Desired	Opposite	Neutral	Proportion	Remainder
Weight	73	−0.025	31	23	19	42%	35% *
BMI or % body fat	37	−0.023	16	12	9	43%	47%
Cholesterol	30	−0.079	15	4	11	50%	41%
Exercise	95						
Diet	58						
Strength	35						
Lipid profile	10						
Vitamins	8						
Blood pressure	10	−0.060	4	4	1	44%	42%
Calcium	5	0.090	3	1	1	60%	34%
Glucose	5	−0.030	3	1	1	60%	42%
Insulin	3	−0.029	1	1	1	33%	19%
Bone density	6						
Stress/anxiety (GAD7)	14	−0.073	9	4	1	64%	49%
Sleep	22	−0.067	13	2	6	62%	50%
Other	23						

The table indicates the number of individuals who prioritized the trait as a target of change (Number), for whom the slope of change was in the desired or opposite direction beyond the absolute value of the indicated threshold (arbitrarily corresponding to one quarter of a standard deviation unit of the slope values), or did not change (neutral). The proportion of the individuals whose change was in the desired direction is also shown, compared with proportion in the remainder of the 162 participants who did not indicate prioritization of the trait (* Fisher’s exact *p* = 0.01, uncorrected for multiple comparisons). Missing cells are either because a simple measure of change is not available, or the sample is too small.

**Appendix Figure A1:**
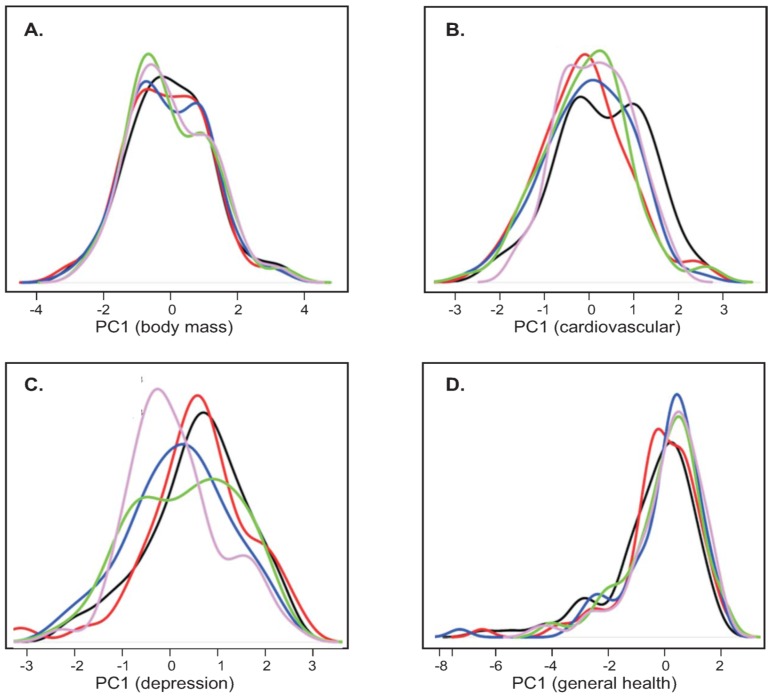
Maintenance of health improvement over three years. Kernel density plots as in Figure 2, but with addition of data from smaller numbers of individuals at Visit 4 (Year 2, green) and Visit 5 (Year 3, pink), showing maintenance of the general trend toward improved health in each case. Frequency distribution curves are for (**A**) PC1 of body mass traits, (**B**) PC1 of cardiovascular traits, (**C**) PC1 of mental health traits, and (**D**) PC1 of general health.

The overall trends for health improvement are encouraging; however follow-up examination of the full cohort will be required to see the long term effect of the program and to quantify the tendency of participants to maintain more healthy lifestyles. We nevertheless performed preliminary analysis of longitudinal data for 83 participants who had completed three year follow-up. As projected in kernel density plots in Appendix Figure A1, the improvement of most of the traits was maintained for at least three years of follow-up. Notably, a continuously decreasing trend in Beck depression score and associated improvement in mental health of the participants from baseline to Year 2 (green) and Year 3 (pink) follow-up was observed. A similar trend was also seen for physical health of the participants. Though a larger dataset for three year follow-up will solidify these conclusions, the analyses reported here suggest that the findings of one year follow-up may be extrapolated for longer terms, highlighting the potential of programs such as CHDWB for the prevention of chronic disease.

### 2.2. Discussion

#### 2.2.1. Central Findings

We present here a report of the influence that participation in the CHDWB wellness program has on the improvement of participant health and reduction in risk factors for non-communicable disease. Overall, the program showed significant reduction of the burden of NCD risk factors, most notably a reduction in the proportion of individuals at high risk. Though the program enrolled “essentially healthy” individuals from the population, almost half of the participants were at the risk of developing NCDs, particularly for cardiovascular disease. Within just six months of the healthy lifestyle program, participants showed statistically significant improvement in multiple aspects of physical, physiological and psychological health. Body fat percentage, BMI, systolic blood pressure and serum lipid concentrations which are major risk factors for CVDs and most of the NCDs showed reductions in just the initial six months and this continued for at least one year. Correspondingly, the Framingham risk score for 10 years risk of CVD also reduced significantly.

Despite significant reductions in BMI, blood pressure and lipids which are risk factors for glucose imbalance and type 2 diabetes, no change in glucose metabolism related traits or FRS (Diabetes) was observed. This might be because very few individuals had higher glucose levels to start with at the baseline (N = 9 with glucose >100 mg/dL) and thus power was not sufficient to capture a meaningful improvement. Similar to the trends we observed for other traits, those individuals with high glucose levels did reduce glucose and insulin levels over time when compared with other random sets of an equal number of individuals.

**Figure 5:**
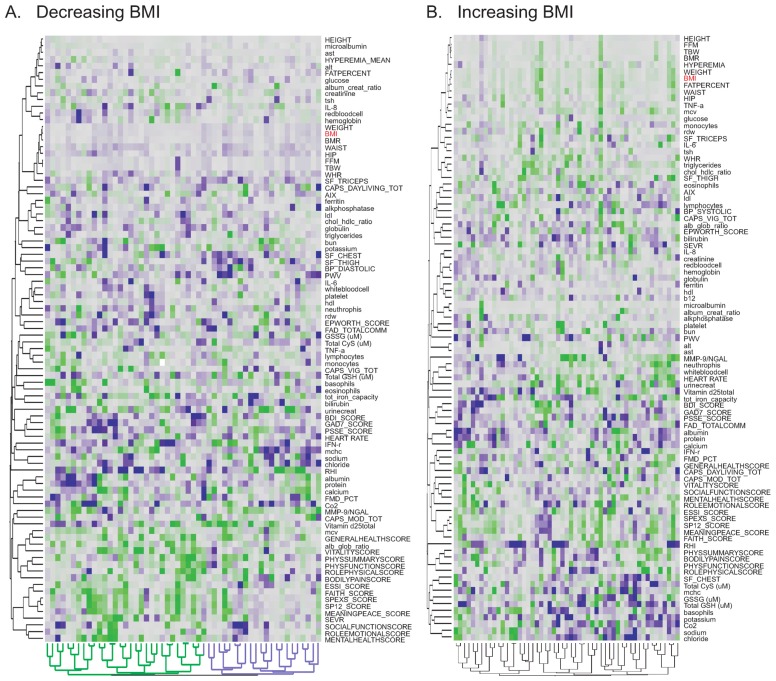
Two-way hierarchical clustering of 91 traits. (**A**) Clustering based on Ward’s method for 53 people whose BMI reduced (slope less than −0.023 over first three visits), showing two main groups of 30 people whose general health scores improve (positive slopes, green) and 23 people who do not show any common trends for other traits; (**B**) Clustering for 53 people whose BMI increased (slope greater than 0.023 over first three visits), showing no strong sub-groups.

We also established that the health improvements were more likely to occur in those individuals at highest risk for each trait. Since these are themselves correlated, we observe a general trend for the least healthy individuals to show the most marked responses for multiple traits, generally in the desired direction. Indeed, among the 53 individuals who showed the greatest reduction in BMI, two clusters are observed (Figure 5A). Just over half of these individuals show coordinated improvement in almost all of their general health and vitality measures. No such pattern is observed either for the remainder of the reduced-BMI individuals, whose apparent health improvement may consequently actually in part be attributed to the phenomenon of regression to the mean, namely an artifact of inflated measures at baseline due to measurement error. With just three time-points in the current longitudinal analysis there is little power to assess significance of individual slopes, but the coordinated responses are only seen for health improvement. That is to say, there is no overall trend toward worsening of health for just a subset of the cohort.

#### 2.2.2. Efficacy of the Lifestyle Intervention

Aside from individual benefits, a wellness program such as the CHDWB should be evaluated with respect to the potential economic benefits. These can be modeled as a function of four parameters: the fraction of individuals who show significant benefits, the magnitude of those benefits, the economic impact of the improvement in health, and the size of the sample. Overall statistics reported in Table 1 and Figure 2 imply study-wise improvements, but closer examination of data supporting Figure 3 and Figure 4 indicates that these gains are apparently restricted to a relatively small fraction of the study, between 10% and 15%. Consequently, from an economic perspective, the reduced healthcare expenses for these individuals must offset the participant costs for the entire sample. We assume that the 5% to 10% of individuals for whom some health indicators trend in the wrong direction would have shown the same changes without participation; whether the program prevented another fraction from following a similar trajectory could only be evaluated relative to a control cohort, which is not available.

Approximating annual costs of participation in the full CHDWB program including health partner services and all medical tests of $1000 per individual participant, the health savings due to health improvement for 10% of the participants would need to be in excess of $10,000 per year for each of those whose health does improve. This number would include avoidance of expensive in patient services, long-term care for chronic conditions (diabetes, kidney disease), medication, and gains in employee-performance (absenteeism, productivity). Obesity alone is estimated to cost the US healthcare system approximately $150 billion annually, or $500 per person and over $5000 per obese individual [8]. Consequently, it is conceivable that participation costs for some individuals may approximate the actual expenses due to chronic non-communicable disease.

It is in theory possible to evaluate the health-care costs associated with people in each clinical category, and to compute savings from the difference for individuals who transition into a new category [9]. This assumes that those individuals adopt the cost profile of the new group, rather than maintaining the legacy of their prior ill-health. In any case, most individuals would be unlikely to see financial benefits of participation, at least in the short term, since the health benefits are minor and mostly deferred to the future. Our data suggests though that the strongest improvements are observed for the highest risk individuals, and hence that those individuals are the most likely to benefit directly from a personalized health discovery program such as this. This may imply that it is most cost-effective for employers and/or insurers to pay for the participation of high risk employees.

#### 2.2.3. Mechanism of Health Improvement

Our analyses do not provide any insight into the reasons for the improvement in general health parameters that we document. The interpretation that health partners are effective at providing counsel, and directing participants to focus on those aspects of their health that they are most concerned to prioritize, is only weakly supported by the data in Table 3. The number of participants who emphasized mental health (sleep and anxiety) was notably less than those concerned about their weight, but they appear to be more likely to make worthwhile lifestyle changes. There was no significant effect of targeted intervention on BMI or cholesterol, although a much larger sample could provide evidence for a meaningful impact. Unfortunately, we do not have convenient metrics for measuring improvement in diet or exercise, which were the two major behaviors that participants targeted. It is also possible that enrolment in the study provides sufficient self-motivation for participants to act regardless of their contact with health partners.

## 3. Experimental Section

### 3.1. Subject Recruitment

This report includes data for a total of 688 subjects aged 18 to 82 years from the longitudinal cohort of essentially healthy participants recruited as part of the Emory-Georgia Tech Predictive Health Initiative at the CHDWB, located at Emory University Midtown Hospital (Atlanta, GA, USA). The term healthy is not meant to imply a high level of fitness or absence of disease, rather it refers to absence of functional impairment. The participants were recruited either by advertisement or invitation to a random sample of Emory employees. They are broadly representative of Emory employees, covering occupations from janitorial staff to upper administration with roles in healthcare or general academic services, and were free of any known acute illness at the time of recruitment., Participants are taking a wide diversity of medications, but no attempt was made in this study to monitor changes in medication usage or the effect of medication on outcomes. Subjects with uncontrolled or poorly controlled acute or chronic diseases including cardiovascular, endocrine, autoimmune, inflammatory, gastro-intestinal, psychiatric, neurological, musculo-skeletal, infectious, or respiratory disease were excluded. Informed consent was obtained from each participant following protocols approved by the institutional review boards of the two participating institutions, Emory University and the Georgia Institute of Technology.

### 3.2. Measures

Comprehensive health assessments including physical, biochemical and psychological measurements were made at the time of recruitment, along with survey-based collection of information about lifestyle such as diet, physical activity, sleeping pattern, stress level and social support. Self-reported family and personal medical history were also recorded. Physical measurements included anthropometry, fat percentage, bone density, blood pressure, treadmill fitness test and ultrasonic measurements of carotid artery thickness, arterial compliance, and flow-mediated vasodilation (FMD). Biochemical investigation included estimation of serum levels of biomarkers related to glucose and lipid metabolism, oxidative stress, endocrine, immune and inflammatory functions. General, physical and mental health of the participants were assessed through a set of standard questionnaires and, based on these, physical summary score, physical function score, mental summary score, depression index and mental summary score were obtained as indices of the state of physical and mental health. A detailed description of the recruitment procedure and health assessments was provided previously [4,10]. Framingham risk scores for eight year risk of diabetes [FRS (Diabetes)] and 10 year risk of cardiovascular diseases [FRS (CVD)] were calculated as described previously [6,11]. Individuals with BMI > 30 kg/m^2^ were classified as obese. Diabetes mellitus was defined as per American Diabetes Association criteria [12] while hypertension was defined according to the Seventh Report of the Joint National Committee on the Prevention, Detection, Evaluation, and Treatment of High Blood Pressure (JNC 7) [13]. Self-reported medication usage is available, and a majority of participants reported taking drugs or supplements, many also before enrolment and including most individuals with diabetes, known hypertension or high cholesterol. Owing to the diversity of medications and relatively small sample with a wide age range, no attempt was made to statistically evaluate the effect of modification of medication usage on health outcomes.

### 3.3. Follow-up Visits

All the participants were invited for evaluation after six months and 12 months, and approximately at one year intervals thereafter. As of July 2013, a total of 502 participants had completed six-month evaluation (Visit 2), 382 had completed one-year evaluation (Visit 3), 232 had completed two-year evaluation (Visit 4) and 83 had completed three-year evaluation (Visit 5). All the measurements including anthropometric, biochemical and clinical examinations mentioned above were made at all the visits as per the protocol at baseline. Surveys of mental health, diet and exercise were conducted either during the visit or online in the days prior to the visit. Immediately after each visit, participants met with one of six professional “health partners”, with whom they reviewed all of their data, discussed priorities, and recalibrated health goals for the next year. The majority of participants continued to communicate with their health partner, who as far as possible remained the same throughout the study, at least monthly either by email or telephone, thereby receiving updates on biochemical tests, as well as ongoing motivation and counsel.

### 3.4. Statistical Analyses

Descriptive data are presented as mean ± standard deviation for continuous variables or percentages for categorical outcomes, unless otherwise mentioned. The continuous variables were log transformed and converted to z-scores before analysis. Change in each trait was calculated as the difference of each visit level relative to the baseline for the participant. Significance of the changes between baseline and six months follow-up and between baseline and one year follow-up was tested using paired sample Wilcoxon signed rank tests. Unpaired sample Wilcoxon signed rank tests were performed to compare the medians of the cohort at different visits. Univariate regression analysis was performed both to estimate the sign and magnitude of the slope of change in each measure over three, four or five visits, and to investigate the relationship between the variables. Correlations between continuous variables were investigated by Pearson’s correlation. Principal components analysis was performed to reduce the number of factors. The first principal components for CVD related traits (systolic blood pressure (BP), augmentation index (AIX), and pulse wave velocity (PWV)), obesity related traits (body mass index (BMI) and percent body fat (%BF)), physical and mental health (physical summary score, general health score, mental health score, social function scores), and mood (Beck depression index (BDI), generalized anxiety (GAD7), sleep (Epworth scale), perceived social self-efficacy and (PSSE)) were generated and used as outcomes in the correlation and regression analyses. Hierarchical clustering of slopes was performed using Ward’s method. All statistical analyses were performed using R or JMP Genomics v5 (SAS Institute, Cary, NC, USA).

## 4. Conclusions

Participation in our wellness program has resulted in a significant number of people adopting minor changes in their eating, exercise, and stress management that lead to health improvements. The growing quantified self-movement [14] is built on the principle that self-knowledge and goal-setting is an effective tool for health promotion, and numerous online social network communities such as SparkPeople, OneCare.me, and mdRevolution, are building businesses around this theme. They rely on communications from peers, families, and automated text-messaging to bolster self-report, providing the motivation to stick to wellness programs. There is much to be learned about the depth of self-knowledge that is required to motivate change in health behaviors, the efficacy of different modes of communication, and the economic incentives to individuals and employers. Modest as the gains reported here may be, they appear to be substantial at least for some individuals, strongly supporting the contention that clinical self-knowledge will be at least as important as genomic self-knowledge for the growth of personalized medicine.

## Appendix

**Appendix Table A1:** Demographic and clinical characteristics of CHDWB cohort at first visit.

Trait	Measurement
N (M/F)	668 (217/451)
Age (years)	48.3 ± 10.9
Ethnicity (% Cau/AA/Asian)	72/23/5
Obese (%)	28.4
Diabetes (%)	3.6
Hypertension (%)	22.1
Fat percent	33.1 ± 9.9
Diastolic BP (mmHg)	76.3 ± 10.8
Systolic BP (mmHg)	120.9 ± 15.9
Glucose (mg/dL)	89.8 ± 17.9
hsCRP (mg/dL)	0.43 ± 0.50
Total cholesterol (mg/dL)	194.6 ± 36.3
HDL-C (mg/dL)	63.7 ± 17.9
LDL-C (mg/dL)	110.6 ± 31.9
Triglycerides (mg/dL)	102.4 ± 59.9
25-hydroxyvitamin D (ng/mL)	30.5 ± 12.2
FRS (CVD)	5.8 ± 5.9
FRS (Diabetes)	2.0 ± 4.0
General Health Score	52.6 ± 8.0
Mental Health Score	51.7 ± 8.0

Data presented are mean ± standard deviation or percentage. HDL-C: high density lipoprotein-cholesterol; LDL-C: high density lipoprotein-cholesterol; FRS: Framingham risk score; CVD: cardiovascular disease.

**Appendix Table A2:** Correlation between slopes of traits.

Trait 1	Trait 2	PCC	*p* Value
BMI	Systolic B.P.	0.18	3.4 × 10^−4^
BMI	Cholesterol: HDL-C	0.18	4.1 × 10^−4^
BMI	FRS (CVD)	0.18	3.7 × 10^−4^
BMI	FRS (Diabetes)	0.22	2.0 × 10^−5^
BMI	General Health Score	−0.20	9.2 × 10^−5^
BMI	Insulin	0.23	5.6 × 10^−4^
BMI	SEVR	−0.21	2.7 × 10^−5^
BMI	Triglycerides	0.21	5.2 × 10^−5^
Calcium	Vitamin B12	0.19	2.4 × 10^−4^
Calcium	25-hydroxyvitamin D	0.21	4.2 × 10^−5^
Calcium	LDL-C	0.24	3.4 × 10^−6^
Calcium	Total Cholesterol	0.28	1.7 × 10^−8^
FRS (CVD)	Diastolic B.P.	0.34	1.0 × 10^−11^
FRS (CVD)	Cholesterol: HDL-C	0.42	3.2 × 10^−17^
FRS (CVD)	LDL-C	0.37	1.9 × 10^−13^
FRS (CVD)	Total Cholesterol	0.37	1.8 × 10^−13^
FRS (CVD)	Triglycerides	0.29	1.8 × 10^−8^
Glucose	HDL-C	−0.17	9.8 × 10^−4^
Glucose	Insulin	0.24	3.6 × 10^−4^
Glucose	Triglycerides	0.19	1.8 × 10^−4^
HDL-C	PWV	0.19	2.9 × 10^−4^
IL-8	Total GSH	0.23	1.3 × 10^−5^
Total Body Fat	Total Cholesterol	0.22	1.9 × 10^−4^
Total Body Fat	Triglycerides	0.21	5.6 × 10^−4^
Triglycerides	Cholesterol: HDL-C	0.54	2.6 × 10^−30^
Triglycerides	FRS (Diabetes)	0.31	1.1 × 10^−9^
Triglycerides	Insulin	0.30	7.0 × 10^−6^
Triglycerides	Total Cholesterol	0.29	9.2 × 10^−9^
